# A Blockchain-Enabled Multi-Authority Secure IoT Data-Sharing Scheme with Attribute-Based Searchable Encryption for Intelligent Systems

**DOI:** 10.3390/s25195944

**Published:** 2025-09-23

**Authors:** Fu Zhang, Xueyi Xia, Hongmin Gao, Zhaofeng Ma, Xiubo Chen

**Affiliations:** 1 School of Cyberspace Security, Beijing University of Posts and Telecommunications, Beijing 100876, China; zhangfu@bupt.edu.cn (F.Z.); xueyixia@bupt.edu.cn (X.X.); xb_chen@bupt.edu.cn (X.C.); 2 Beijing University of Posts and Telecommunications-China Mobile Communications Group Co., Ltd. Joint Institute, Beijing 100876, China; gaohongmin@chinamobile.com; 3 China Mobile Information Technology Co., Ltd., Beijing 102206, China; 4 Beijing Key Laboratory of Trusted Regulation and Governance for Artificial Intelligence, Beijing 102206, China

**Keywords:** Internet of Things, blockchain, multi-authority, attribute-based searchable encryption, security

## Abstract

With the advancement of technologies such as 5G, digital twins, and edge computing, the Internet of Things (IoT) as a critical component of intelligent systems is profoundly driving the transformation of various industries toward digitalization and intelligence. However, the exponential growth of network connection nodes has expanded the attack exposure surface of IoT devices. The IoT devices with limited storage and computing resources struggle to cope with new types of attacks, and IoT devices lack mature authorization and authentication mechanisms. It is difficult for traditional data-sharing solutions to meet the security requirements of cloud-based shared data. Therefore, this paper proposes a blockchain-based multi-authority IoT data-sharing scheme with attribute-based searchable encryption for intelligent system (BM-ABSE), aiming to address the security, efficiency, and verifiability issues of data sharing in an IoT environment. Our scheme decentralizes management responsibilities through a multi-authority mechanism to avoid the risk of single-point failure. By utilizing the immutability and smart contract function of blockchain, this scheme can ensure data integrity and the reliability of search results. Meanwhile, some decryption computing tasks are outsourced to the cloud to reduce the computing burden on IoT devices. Our scheme meets the static security and IND-CKA security requirements of the standard model, as demonstrated by theoretical analysis, which effectively defends against the stealing or tampering of ciphertexts and keywords by attackers. Experimental simulation results indicate that the scheme has excellent computational efficiency on resource-constrained IoT devices, with core algorithm execution time maintained in milliseconds, and as the number of attributes increases, it has a controllable performance overhead.

## 1. Introduction

With the deep integration and rapid development of technologies such as artificial intelligence, big data, and cloud computing, intelligent systems have become the core driving force for the new round of technological revolution and industrial transformation. By simulating human cognition and decision-making processes, intelligent systems achieve perception, analysis, learning, and autonomous responses to complex environments, and they are widely applied in intelligent manufacturing, smart healthcare, intelligent transportation, and other fields. As the “senses” and “nerve endings” of intelligent systems, the Internet of Things (IoT) undertakes the key functions of real-time data collection, device interconnection, and edge control, serving as the fundamental infrastructure for the deep integration of the physical world and the information space. The evolution of IoT technology has greatly enhanced the perception capabilities and coverage of intelligent systems. As a vast and complex ecosystem, the IoT encompasses multiple dimensions, ranging from hardware devices to software platforms, and from data collection to intelligent analysis. With its remarkable convenience, powerful integration, and flexible scalability, the IoT demonstrates unique advantages [1]. With the acceleration of digital transformation, the IoT has been extensively implemented in various sectors, including telemedicine, smart communities, and the Internet of Vehicles (IoV). It not only significantly improves the operational efficiency and service quality of various industries but also accelerates the digital technological transformation of society to a certain extent [2].

According to the GSMA, the global scale of IoT connections will surpass the 6.3 billion recorded in 2017, reaching a staggering 25.2 billion [3]. The exponential growth of network connection nodes has expanded the attack exposure of IoT devices, and security issues at the device end need to be urgently addressed. To reduce costs and power consumption, IoT terminal devices are typically equipped with weak computing capabilities and low levels of intelligence, making it difficult for them to perform complex data encryption operations. Meanwhile, IoT devices have a long life cycle, and many of them have stopped updating, leaving limited room for upgrades. In this scenario, complex security policies are challenging to implement on a significant number of low-power devices due to their limited computing and storage capabilities and lack of the ability to deal with new types of attacks. A report shows that 98% of IoT endpoints transmit data without encryption, and 83% of medical imaging devices in the United States have discontinued security updates. In addition, many IoT devices transmit data in plain text and lack mature authorization and authentication mechanisms, which can easily lead to data leakage and unauthorized access. However, assigning complex computing tasks to edge computing devices can also trigger data security risks and threaten user privacy. Without compromising control over data, the cloud server must store and process the vast distributed data collected from the sensors involved in the intelligent system, and it is difficult for traditional data-sharing solutions to meet its security requirements [4].

Attribute-based encryption (ABE) technology ensures data confidentiality while also enabling data owners to formulate flexible access control policies. However, relying on a single attribute authority may encounter performance limitations and the risk associated with having a single point of failure. Therefore, we design a multi-authority attribute-based encryption method to enhance the system’s reliability and flexibility. In addition, considering that cloud computing platforms typically provide computing and storage services to multiple users, the issue of how to quickly retrieve the required data from massive data needs to be addressed. For this reason, we suggest a searchable encryption scheme that is attribute-based to enhance the efficacy of information retrieval. In the current cloud computing environment, IoT data are at risk of tampering and misuse. To safeguard the credibility of search outcomes and the integrity of data, we implement blockchain technology. The immutability and transparency of blockchain provide a strong guarantee for data integrity, while the smart contract function can automatically execute and verify data operations.

In summary, we present a blockchain-based multi-authority IoT data-sharing scheme using attribute-based searchable encryption for intelligent systems (BM-ABSE). In this scheme, individuals with constrained resources can delegate the decryption tasks to a cloud computing platform by using the attribute decryption key, thereby reducing their own computational burden to adapt to the resource-constrained computing environment. The hash values derived from plaintext data will be recorded on the blockchain for easily verifying the computation results of the cloud platform. Moreover, the alignment procedure between search trapdoors and indexes is executed via smart contracts, providing a guarantee for the credibility of search results of ciphertext data. The proposal in the paper aims to achieve a safe, reliable, and efficient data-sharing environment for the IoT, highlighting the following major contributions:

- We propose a multi-authority attribute-based encryption scheme, BM-ABSE, employing LSSS. By decentralizing management responsibilities, it enhances the reliability and flexibility of the system, prevents system paralysis caused by the failure of a single administrator, and strengthens the robustness of the system.
- We adopt blockchain to replace the trusted certificate authority (CA) in traditional CP-ABSE, establishing a trustless collaborative mechanism between blockchain and attribute-based encryption. This approach eliminates reliance on a single center authorization and solves the challenge of rapidly locating required information in massive encrypted data. By automatically executing and verifying data operations through smart data, we effectively guard against malicious behaviors of cloud servers, thereby enhancing the reliability of ciphertext search.
- We conduct a thorough security analysis and formally prove that the proposed scheme achieves static security and indistinguishable under chosen keyword attack (IND-CKA) in the random oracle model. In addition, in the performance evaluation and simulation experiments, we employ three elliptic curves to achieve different security levels, analyzing and confirming the reliability and efficiency of our scheme.

The subsequent sections of this work are organized as follows: Section 2 analyzes the related work. Section 3 presents the theoretical foundations of adopted cryptography and definitions of relevant notations used in this paper. Section 4 presents the system overview, threat model, and security definitions. In Section 5, our proposed scheme is elucidated in depth. A comprehensive security analysis of our methodology is presented in Section 6. Section 7 discusses the performance evaluation and simulation experiments of the system. Ultimately, we conclude the paper in Section 8.

## 2. Related Work

### 2.1. ABE-Based Searchable Encryption

In the environment of IoT and cloud computing, a rising volume of enterprise and user data is uploaded to cloud servers. However, these data and associated resources in cloud computing rely on unreliable network communication and semi-trusted cloud storage services [5]. To guarantee the security of information, a current efficient method involves the data owner encrypting the data before uploading it to server, while the data user decrypts it when needed.

Searchable encryption (SE) technology is developed to enable data users to have control over ciphertexts in cloud storage, that is, to search encrypted data without decrypting it. Boneh et al. developed the public key encryption with keyword search (PEKS) scheme [6]. Ref. [7] presented a forward secure searchable symmetric encryption (FSE) scheme using only symmetric cryptography, which supports dynamic file insertion and deletion, guarantees forward privacy protection, achieves sublinear search complexity, and attains partial backward privacy through a space reclamation mechanism after deletion. Li et al. designed a lightweight encrypted phrase search scheme (LPSSE) that can efficiently accomplish the phrase search in encrypted documents in the cloud through only one round of interaction, taking into account both lightweight and non-adaptive security [8]. Caldarola et al. proposed a consensus protocol that balances privacy, fairness, and efficiency, utilizing neural networks, elliptic curve cryptography (ECC), and verifiable cryptographic lotteries (VCLs) to achieve fair node selection and privacy protection [9]. However, fine-grained access control in multi-user settings cannot be realized using traditional public key cryptography and a symmetric cryptosystem.

To realize fine-grained access control and efficient search capabilities while simultaneously guaranteeing data privacy and confidentiality, researchers have introduced attribute-based searchable encryption approaches that integrate the concepts of ABE. Khader systematically defined attribute-based searchable encryption (ABSE) primitive, which integrates the concept of ABE into public-key searchable encryption (PKSE), allowing the sender to control both decryption and search permissions with attribute policies [10]. Zheng et al. initially presented the verifiable attribute-based keyword search (ABKS) method, allowing authorized users to authenticate the accuracy of cloud-returned results after conducting keyword searches on encrypted data stored in the cloud, all without needing to interact with the data owner [11].

In recent years, driven by the dual imperatives of data security and searchability, the application of ABSE schemes in industrial IoT devices has gained significant momentum. Ali et al. proposed a verifiable online/offline multi-keyword search (VMKS) approach, which enables resource-constrained IIoT nodes to conduct fine-grained, thresholded keyword searches in the cloud and verify results correctness without downloading the entire dataset, thus balancing efficiency and security [12]. For industrial IoT, Zhou et al. designed a device-oriented keyword searchable encryption (Do-KSE) scheme to realize “device-centric” rather than “user-centric” data localization and status monitoring [13]. Yin et al. embedded access policies within encrypted inverted indexes to achieve fine-grained keyword retrieval authority control for ciphertext data in cloud-assisted IIoT scenarios [14]. Nevertheless, the ABSE schemes that are currently in place are dependent on a single authority to issue keys and policies, which leads to risks of single-point failure and cross-domain mutual exclusion and also make it difficult to meet the demand of large-scale data sharing in the IoT. We introduce an IoT data-sharing scheme called BM-ABSE to address this issue, which delivers dynamic access control and a verifiable fine-grained keyword search.

### 2.2. Multi-Authority Attribute-Based Encryption

In single-authority ABE approaches, the key distribution work is entirely undertaken by a sole CA. When the user scale or attribute dimension expands dramatically, the computing and communication load that CA needs to complete increases exponentially, making it a critical performance bottleneck. Moreover, the system security must be forced to build on the assumption that the CA is unconditionally trustworthy. Once the CA is compromised or malicious, it can arbitrarily forge user keys, tamper with access policies, and even decrypt all the historical ciphertexts offline through the global master key, thereby triggering a disaster of key escrow and a single point of failure. Conversely, in a multi-authority ABE (MA-ABE) system, the attribute space is split into several mutually invisible subsets according to the domain or organizational boundaries. Each attribute authority independently governs its own master key and attribute private key fragments within its domain. The attributes are managed by autonomous attribute authorities, and the collusion of one or more attribute authorities cannot undermine the security of the entire system. In real-world scenarios, different organizations need to apply different strategies to share information; for example, hospitals belong to different legal entities, cloud service providers have separate compliance domains, and equipment vendors maintain independent identity repositories. A single authorization authority cannot satisfy the governance requirements of “each governing its own attributes, policies can be cross-domain”. Consequently, multiple authorization authorities are needed to control the attributes of all users separately, and constructing a secure and reliable MA-ABE system is the key to solving the above problems.

To mitigate the inherent constraints of a singular authority on efficacy and reliability, the development of MA-ABE can be characterized as a progression through four paradigms: centralized, weakly decentralized, fully decentralized, and on-chain governance. The corresponding core contradiction is the degree of decentralization, security, functionality, and governance cost. In 2007, Chase implemented the first MA-ABE scheme, where a central authority is tasked with creating public and private keys for various attribute authorities, while the latter distribute keys and manage attributes [15]. Compared with the single-authority scheme, resisting collusion among users becomes more challenging. Chase introduced a global identity (GID) for each user, binding private keys tightly to GID so that colluding users still cannot decrypt ciphertexts. However, malicious authorities in this scheme can trace users’ GIDs to aggregate their attributes, thus violating users’ privacy. In 2009, Chase et al. presented an MA-ABE scheme without a centralized authorization center, which eliminates the need for a reliable central authority through the use of a distributed pseudo-random function (PRF) [16]. In particular, the user obtain its private key by using an anonymous key distribution protocol, during which the authorities are unable to obtain any relevant information regarding user’s GID, thus resolving the privacy preservation issue. Liu et al. constructed the first MA-ABE model without a central authorization center under the standard model [17]. In their scheme, the system must be initialized by a combination of authorities during the setup phase. In addition, if an attribute needs to be added to the system, multiple authorities must collaborate to reconfigure the system. Lewko et al. developed an MA-ABE approach within the random oracle model using composite-order bilinear groups [18]. This scheme supports monotone span programs but is restricted to the attribute of a small universe, indicating that the quantity of attributes in the system is directly proportional to the magnitude of the public parameters.

At present, MA-ABE has been widely implemented in the realm of cloud and edge computing. Zhou et al. introduced a multi-authority CP-ABE access control framework for multi-cloud storage environments [19]. By introducing an attribute mapping mechanism, it resolves cross-cloud policy conflicts and achieves decentralized and semantically consistent fine-grained data sharing. However, the scheme still relies on a centralized cloud service broker (CSB) as the global root of trust, which fails to completely eliminate the risks of single points of failure and privacy leakage. Huang designed a multi-authority CP-ABE scheme for a large attribute domain environment in edge computing that combines an offline reusable ciphertext pool and online lightweight encryption with outsourced decryption by an edge server, reducing decryption and encryption overhead on the user side to a single exponential operation while preserving static security and high efficiency for resource-constrained IoT devices [20]. Guo et al. demonstrated a multi-authority CP-ABE method without a pairing operation, which leverages the construction of an elliptic curve discrete logarithm and cloud-edge outsourcing to compress terminal encryption and decryption overhead to a constant number of exponential operations, simultaneously achieving fine-grained policy, collusion resistance, and lightweight performance [21].

## 3. Preliminary

This section provides an overview of the fundamental cryptographic building blocks. The symbols employed in our paper are illustrated in Table 1.

### 3.1. Bilinear Pairing

The bilinear pairing maps two group elements from an elliptic curve group to an element in a multiplicative group while preserving their isomorphic properties. The scheme in this paper is theoretically based on symmetric bilinear pairings, which are denoted as $e:G\times G\to G_{T}$, where G and $G_{T}$ are cyclic groups of prime order *p*. With the generators (u,v) of G, the bilinear pairing has the following properties:

- Bilinearity: For all u,v∈G and $a,b\in Z_{p}$, it holds that $e\left(u^{a},v^{b}\right)=e{\left(u,v\right)}^{ab}$, where $Z_{p}=\left\{0,1,2,\ldots ,p-1\right\}$;
- Non-degeneracy: If e(u,v)=1, then either u=1 or v=1;
- Computability: For any u,v∈G, an efficient algorithm exists to compute e(u,v).

### 3.2. Access Structure

Let a set of participants be $P=\{p_{1},p_{2},\ldots ,p_{n}\}$, where each $p_{i}$ represents a user or entity in the system. A set $C\subseteq 2^{P}$ is monotonic when it satisfies the following: for any two sets X and Y, if X∈C and X⊆Y, then Y∈C.

**Definition** **1.** *A monotone access structure is defined as $C\subseteq 2^{P}\setminus \left\{\emptyset \right\}$; that is, C includes all non-empty subsets of P. This guarantees that every set in C contains at least one participant, thus avoiding the special case of an empty set. The sets in C are called authorized sets. Otherwise, they are called unauthorized sets.*

### 3.3. Linear Secret Sharing Scheme

Assume that $P=\{P_{1},P_{2},\ldots ,P_{n}\}$ is a set of participants. A secret sharing scheme Π on P is linear if and only if the following two conditions are satisfied:

1. The secret shares *s* held by all participants can form a vector on $Z_{p}$.
2. There exists a secret share generation matrix $M_{l\times n}$; each row $M_{i}$ of the matrix can be mapped to a participant $p_{i}$ through a function ρ(i). Define a vector $v=(s,v_{2},\ldots ,v_{n})$, where $s\in Z_{p}$ is the secret value to be shared, and $v_{2},\ldots ,v_{n}$ are randomly selected from $Z_{p}$. The secret share of *s* on each row of the secret generation matrix can be represented as $\varphi_{i}=M_{i}\cdot v$; that is, the secret share held by each participant ρ(i) is $\varphi_{i}$.

The linear secret sharing scheme (LSSS) features linear reconfiguration. Suppose that an LSSS Π represents an access structure, let S⊆P represent an authorized attribute set, and the index set I⊂{1,…,l} is defined as I={i:ρ(i)∈S}. Based on linear reconstruction properties, there exists a collection of constants ${\left\{\omega_{i}\in Z_{p}\right\}}_{i\in I}$, such that $s=\sum_{i\in I}\omega_{i}\varphi_{i}$. Moreover, these constants can be determined in polynomial time. For each unlawful set, it is infeasible to identify a set of constants that fulfill the criterion.

## 4. System Overview and Security Requirements

### 4.1. System Model

**Figure 1:**
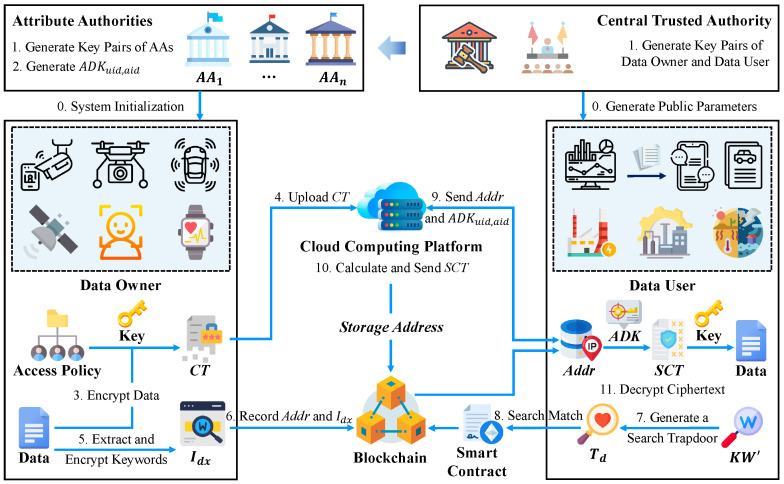
System model of BM-ABSE.

The BM-ABSE consists of several roles: the central trusted authority, a set of attribute authorities, the data user, the data owner, and the cloud computing platform. The central trusted authority is a completely trusted entity that is responsible for the system initialization and the establishment of public parameters, while attribute authorities oversee separate sets of attributes and create corresponding attribute-related keys. The system model is depicted in Figure 1.

### 4.2. Threat Model

Our threat model assumes the reliability of attribute authorities and distributes attribute decryption keys to authenticated individuals through secure channels. IoT data users might be interested in the ciphertexts stored on the chain and may collude with unauthorized parties to obtain plaintext information. To evaluate the security of the proposed approach, we describe two categories of attackers:

- The initial kind seeks to cooperate by employing their secret keys to decode material that they are unable to decrypt independently.
- The second kind of adversary concentrates on differentiating among various ciphertexts.

### 4.3. Security Requirements

#### 4.3.1. Static Security

The static security model is utilized to demonstrate the security of the scheme that supports the setting of a multi-attribute authority. Even if attackers can select and corrupt a certain number of attribute authorities after obtaining the public parameters, the encrypted data will not provide them with any further information regarding the plaintext. In this model, the attacker is permitted to corrupt a certain quantity of attribute authorities following the acquisition of the public parameters, and the chosen authorities remain unchanged until the end of the security game. The attacker’s queries are forwarded to the challenger right after receiving the public parameters. Under the static security model, if the advantage of any probabilistic polynomial-time (PPT) attacker A in the following security game with challenger C is negligible, then the BM-ABSE scheme is considered static secure.

1. *Init*: The challenger C runs the Initialization to create public parameters PP and translates them to A.
2. *Query*: First, a group of compromised attribute authorities is specified by A, which is denoted as $C_{AAs}\subseteq AID$. Afterward, the following queries are executed by A:
  (a) A specifies a set of uncompromised attribute authorities, denoted as $N_{AAs}$, and queries the public keys $MPK_{\theta }$ of this authorities, where $C_{AAs}\cap N_{AAs}=\emptyset$.
  (b) A submits an attribute set $\left\{uid,S_{uid}\right\}$ to C, in which it is required that this set must not be under the control of the compromised attribute authorities. Then, A queries the attribute decryption key $ADK_{uid,aid}$ of $S_{uid}$.
  (c) A presents two plaintexts $m_{0},m_{1}\in G_{T}$ of the same length along with an access structure $\left(A^{*},\delta^{*}\right)$ to C.
3. *Response*: C selects a random plaintext $m_{b},b\in \left\{0,1\right\}$, and replies to A’s queries according to the following process:
  (a) C executes the AuthSetup to return the public key $MPK_{\theta }$ of an uncompromised attribute authority.
  (b) C executes the ADKGen to return the attribute decryption key $ADK_{uid,aid}$ corresponding to the attribute set $\left\{uid,S_{uid}\right\}$.
  (c) C executes DataEncrypt to return the ciphertext $CT^{*}$ of $m_{b}$.
4. *Guess*: A produces its guess $b^{\prime }\in \left\{0,1\right\}$. The probability advantage of A to guess correctly is defined as
  (1) $$Adv_{A}^{static}\left(1^{\theta }\right)=\left|2Pr\left[b^{\prime }=b\right]-1\right|.$$

#### 4.3.2. IND-CKA Security

This security model is used to evaluate whether an attacker can distinguish different ciphertexts under a chosen keyword attack. Even if attackers can select specific keywords and obtain the corresponding ciphertext, they are unable to acquire any supplementary information regarding the keywords from it. Under the IND-CKA security model, the BM-ABSE scheme is deemed IND-CKA secure if the advantage of any probabilistic polynomial-time (PPT) adversary A in the subsequent security game with challenger C is insignificant.

1. *Init*: The Initialization is executed by the challenger C to produce public parameters PP, which are then transmitted to A.
2. *Phase 1*: A submits a set of keywords and issues a search trapdoor query to C. C executes the TrapGen to output the search trapdoor Td and transmits it to A.
3. *Challenge*: A randomly selects two keywords of the same length $kw_{0},kw_{1}$ and submits them to C. In *Challenge*, keywords that were submitted in *Phase 1* are not permitted. The KeywordEncrypt function is executed by C to arbitrarily encrypt one of the keywords $kw_{b},b\in \left\{0,1\right\}$, and it sends the data index $I_{dx}$ back to *A*.
4. *Phase 2*: A can issue any of the queries from *Phase 1*; the keywords that were submitted in the *Challenge* are not eligible for submission.
5. *Guess*: A generates its estimate of $b^{\prime }\in \left\{0,1\right\}$. The ability of A to accurately predict is defined as
  (2) $$Adv_{A}^{IND-CKA}\left(1^{\theta }\right)=\left|2Pr\left[b^{\prime }=b\right]-1\right|.$$

## 5. Details of Our Proposed Scheme

### 5.1. System Initialization

- $Initialization(1^{\lambda })\to PP$: The central trusted authority selects two cyclic groups G and $G_{T}$ of prime order *p* with g∈G as a generator of group G. Then, it creates a bilinear map $\hat{e}:G\times G\to G_{T}$ and chooses three hash functions $H:{\left\{0,1\right\}}^{*}\to Z_{p}^{*}$, $H_{0}:GID\to G$, and $H_{1}:U\to G$. Randomly select an element $\gamma \in Z_{p}^{*}$ and establish $PP=\{\hat{e},G,G_{T},g,g^{\gamma },p,H,H_{0},H_{1}\}$.
- $AuthSetup\left(PP,\theta \right)\to \left(MPK_{\theta },MSK_{\theta }\right)$: Each attribute authority with a unique identifier θ manages a disjoint set of attributes. It randomly selects two exponents $\alpha_{\theta },\beta_{\theta }\in Z_{p}^{*}$ and generates the master secret key pair:
  (3) $$MPK_{\theta }=\left\{\hat{e}{\left(g,g\right)}^{\alpha_{\theta }},g^{\alpha_{\theta }},g^{\beta_{\theta }}\right\},\;MSK_{\theta }=\left\{\alpha_{\theta },\beta_{\theta }\right\}.$$
- $IdKeyGen\left(PP\right)\to \left(PK_{*},SK_{*}\right)$: Every data user with a unique identifier uid or data owner with oid executes this process to generate a pair of keys. The algorithm randomly selects $z_{*}\in Z_{p}^{*}$ and computes $g^{z_{*}}$ where ∗ stands for uid or oid. Then, we output the key pair for the data owner or data user:
  (4) $$PK_{*}=g^{z_{*}},\;SK_{*}=z_{*}.$$
  Using the data user’s public key $PK_{uid}$ and identifier uid, the central trusted authority assigns an attribute set $S_{uid}$ and issues a blockchain transaction $Tx_{reg}=\left\{uid,S_{uid},Timestamp\right\}$.
- $ADKGen\left(PP,MSK_{\theta },PK_{uid},S_{uid,aid}\right)\to ADK_{uid,aid}$: After the data user is successfully registered, each attribute authority runs this process to produce attribute decryption key $ADK_{uid,aid}$ for the attributes it manages. For each attribute $att_{i}\in S_{uid,aid}$, the attribute authority randomly selects $t\in Z_{p}^{*}$ and calculates
  (5) $$\left\{\begin{array}{c} K_{uid,att_{i}}=g^{z_{uid}\alpha_{\theta }}H_{0}{\left(uid\right)}^{\beta_{\theta }}H_{1}{\left(att_{i}\right)}^{t},\\ L_{uid,att_{i}}=g^{t}. \end{array}\right.$$
  The technique ultimately produces the attribute decryption key associated with the attribute set $S_{uid,aid}$ and sends it to the data user for constructing a partially decrypted ciphertext:
  (6) $$ADK_{uid,aid}={\left\{K_{uid,att_{i}},L_{uid,att_{i}}\right\}}_{att_{i}\in S_{uid,aid}}.$$

### 5.2. Data Processing

- $DataEncrypt(PP,MPK_{\theta },F,\left(A,\delta \right))\to CT$: The data owner formulates a data access policy (A,δ), where $A\in Z_{p}^{l\times n}$ is a l×n matrix. The data owner chooses a collection of random numbers $s,x_{2},\ldots ,x_{n},y_{2},\ldots ,y_{n}\in Z_{p}^{*}$ and then constructs two vectors $x=(s,x_{2},\ldots ,x_{n})$ and $y=(0,y_{2},\ldots ,y_{n})$. For each row $A_{i}$ of matrix A, the algorithm calculates $\lambda_{i}=A_{i}\cdot x$ and $w_{i}=A_{i}\cdot y$, respectively, where $\lambda_{i}$ denotes a secret share of the *i*-th row of matrix A and $w_{i}$ represents a share of 0 in the *i*-th row of matrix A. Furthermore, we define two functions δ(i) and ρ(i), where δ(i) maps a row of matrix A to an attribute and ρ(i) maps a row of matrix A to an attribute authority. The data owner chooses a random element $t_{i}\in Z_{p}^{*}$ and computes the components of the ciphertext of the IoT data *F* as follows:
  (7) $$\left\{\begin{array}{c} C_{0}=F\cdot \hat{e}{\left(g,g\right)}^{s},\\ C_{1,i}=g^{\lambda_{i}}g^{\alpha_{\rho (i)}t_{i}},\\ C_{2,i}=g^{\beta_{\rho (i)}t_{i}}g^{w_{i}},\\ C_{3,i}=g^{-t_{i}},\\ C_{4,i}=H_{1}{\left(\delta \left(i\right)\right)}^{t_{i}}. \end{array}\right.$$
  The data owner outputs the IoT data ciphertext CT and subsequently uploads the ciphertext to the cloud computing platform for storage. The upload is in the form of {Hash(CT),CT}, and a successful upload will return a storage address Addr.
  (8) $$CT=(C_{0},{\left\{C_{1,i},C_{2,i},C_{3,i},C_{4,i}\right\}}_{i\in [1,l]}).$$
- $KeywordEncrypt\left(PP,PK_{oid},KW\right)\to I_{dx}$: The data owner extracts a collection of keywords $KW=\{kw_{1},\ldots ,kw_{d}\}$ from the IoT data *F* and then encrypts these keywords. The data owner selects random numbers $\xi ,\xi_{1}\in Z_{p}^{*}$ and calculates the components of the data index $I_{dx}$ as follows:
  (9) $$\left\{\begin{array}{c} W_{1}=g^{z_{oid}\xi },\\ W_{2}=g^{\gamma \xi_{1}},\\ W_{3}=g^{\xi_{1}},\\ W_{i}=g^{\gamma H(kw_{i})\xi }. \end{array}\right.$$
  The data owner outputs the multi-keyword index:
  (10) $$I_{dx}=\left(W_{1},W_{2},W_{3},{\left\{W_{i}\right\}}_{i\in [1,d]}\right),$$
  Finally, the data owner submits the data index $I_{dx}$ and storage address Addr to the blockchain in the form of $\left\{Hash\left(CT\right),I_{dx},Addr\right\}$.

### 5.3. Data Searching

- $TrapGen(PP,SK_{uid},KW^{\prime })\to Td$: The data user generates a search trapdoor using the keywords of interest $KW^{\prime }=\left\{kw_{1}^{\prime },\ldots ,kw_{d^{\prime }}^{\prime }\right\}$. The algorithm randomly chooses $\eta \in Z_{p}^{*}$ and computes the components of the search trapdoor as below:
  (11) $$\left\{\begin{array}{c} T_{1}=g^{\eta z_{uid}},\\ T_{2}=g^{\gamma \eta z_{uid}},\\ T_{3}=\prod_{i=1}^{d^{\prime }}g^{\gamma H(kw_{i}^{\prime })}. \end{array}\right.$$
  The data user outputs the multi-keyword search trapdoor:
  (12) $$Td=(T_{1},T_{2},T_{3}).$$
- $SearchMatch(I_{dx},Td)\to 0/1$: This progress is implemented by a smart contract, taking the data index $I_{dx}$ and search trapdoor Td as inputs. The consensus node executes the smart contract to validate the subsequent equation after receiving a search mechanism from the data user:
  (13) $$\hat{e}\left(T_{3},W_{1}\right)\hat{e}\left(T_{2},W_{3}\right)=\hat{e}\left(T_{1},W_{2}\right)\hat{e}\left(PK_{oid},\prod_{i=1}^{d^{\prime }}W_{i}\right)$$
  Upon equation satisfaction, the process transmits the storage address Addr of the ciphertext to the data user and returns 1. Conversely, the equation produces 0 if it is not valid to indicate a failed search.

### 5.4. Data Decryption

- $SemiDecrypt(PP,PK_{uid},ADK_{uid,aid},CT)\to SCT$: The cloud computing platform runs this algorithm to perform complex decryption operations representing the data user. The algorithm will terminate if the attributes of the data user do not match the access policy. Otherwise, the attribute set satisfying the data access structure (A,δ) is $I={\left\{i:\delta \left(i\right)\in S_{uid}\right\}}_{i\in [1,l]}$. For each element i∈I, the cloud computing center calculates as follows:
  (14) $$\left\{\begin{array}{c} V_{1,i}=\hat{e}\left(PK_{uid},C_{1,i}\right),\\ V_{2,i}=\hat{e}\left(K_{uid,att_{i}},C_{3,i}\right),\\ V_{3,i}=\hat{e}\left(H_{0}\left(uid\right),C_{2,i}\right),\\ V_{4,i}=\hat{e}\left(L_{uid,att_{i}},C_{4,i}\right). \end{array}\right.$$
  (15) $$\begin{array}{cc} tct_{i} & =V_{1,i}\cdot V_{2,i}\cdot V_{3,i}\cdot V_{4,i}\\ \; & =\hat{e}{\left(g,g\right)}^{z_{uid}\lambda_{i}}\hat{e}{\left(H_{0}\left(uid\right),g\right)}^{w_{i}} \end{array}$$
  The cloud computing center calculates a set of constants ${\left\{c_{i}\in Z_{p}^{*}\right\}}_{i\in I}$, such that $\sum_{i\in I}c_{i}\lambda_{i}=s$ and $\sum_{i\in I}c_{i}w_{i}=0$. Combining the constants ${\left\{c_{i}\in Z_{p}^{*}\right\}}_{i\in I}$, the cloud computing center performs the following calculation:
  (16) $$\begin{array}{cc} tct & =\prod_{i\in I}{\left(tct_{i}\right)}^{c_{i}}\\ \; & =\hat{e}{\left(g,g\right)}^{z_{uid}s}. \end{array}$$
  Then, we output the partially decrypted ciphertext:
  (17) $$SCT=\{C_{0},tct\}.$$
  Finally, the cloud computing platform sends SCT to the data user.
- $DataDecrypt(SCT,SK_{uid})\to F$: This algorithm is implemented by the data user, utilizing its private key $PK_{uid}$ and partially decrypted ciphertext SCT as inputs. The algorithm decrypts plaintext data through the following computation:
  (18) $$F=\frac{C_{0}}{tct^{\frac{1}{SK_{uid}}}}.$$

## 6. Security Analysis

### 6.1. Correctness

#### 6.1.1. Search Matching Algorithm

By verifying whether equation holds, the matching of the data index $I_{dx}$ and the search trapdoor Td is achieved.

The expansion of the left-hand side of Equation (13) is as follows:

(19) $$\begin{array}{cc} \mathrm{LHS} & =\hat{e}\left(T_{3},W_{1}\right)\hat{e}\left(T_{2},W_{3}\right)\\ \; & =\hat{e}\left(\prod_{i=1}^{d^{\prime }}g^{rH(kw_{i}^{\prime })},g^{z_{oid}\xi }\right)\hat{e}\left(g^{\gamma \eta z_{uid}},g^{\xi_{1}}\right)\\ \; & =\hat{e}\left(g^{z_{oid}\xi },\prod_{i=1}^{d^{\prime }}g^{rH(kw_{i}^{\prime })}\right)\hat{e}\left(g^{\eta z_{uid}},g^{r_{\xi_{1}}}\right). \end{array}$$

The expansion of the right-hand side of Equation (13) is as follows:

(20) $$\begin{array}{cc} \mathrm{RHS} & =\hat{e}\left(T_{1},W_{2}\right)\hat{e}\left(PK_{oid},\prod_{i=1}^{d^{\prime }}W_{i}\right)\\ \; & =\hat{e}\left(g^{\eta z_{uid}},g^{r_{\xi_{1}}}\right)\hat{e}\left(g^{z_{oid}},\prod_{i=1}^{d^{\prime }}g^{rH(kw_{i})\xi }\right)\\ \; & =\hat{e}\left(g^{\eta z_{uid}},g^{r_{\xi_{1}}}\right)\hat{e}\left(g^{z_{oid}\xi },\prod_{i=1}^{d^{\prime }}g^{rH(kw_{i})}\right). \end{array}$$

When the search keywords are successfully matched, the left and right sides of the equation are equal.

#### 6.1.2. Data Decryption Algorithm

The correctness of this algorithm ensures that the data user whose attributes are satisfied with (A,δ) can obtain partial decrypted ciphertext tct through the cloud computing platform and decrypt it to obtain the IoT data *F*. In particular, calculate Equation (15) first:

(21) $$\begin{array}{cc} tct_{i} & =V_{1,i}\cdot V_{2,i}\cdot V_{3,i}\cdot V_{4,i}\\ \; & =\hat{e}\left(PK_{uid},C_{1,i}\right)\cdot \hat{e}\left(K_{uid,att_{i}},C_{3,i}\right)\cdot \hat{e}\left(H_{0}\left(uid\right),C_{2,i}\right)\cdot \hat{e}\left(L_{uid,att_{i}},C_{4,i}\right)\\ \; & =\hat{e}\left(g^{z_{uid}},g^{\lambda_{i}}g^{\alpha_{\rho (i)}t_{i}}\right)\cdot \hat{e}\left(g^{z_{uid}\alpha_{\theta }}H_{0}{\left(uid\right)}^{\beta_{\theta }}H_{1}{\left(att_{i}\right)}^{t},g^{-t_{i}}\right)\cdot \\ \; & \;\hat{e}\left(H_{0}\left(uid\right),g^{\beta_{\rho (i)}t_{i}}g^{w_{i}}\right)\cdot \hat{e}\left(g^{t},H_{1}{\left(\delta \left(i\right)\right)}^{t_{i}}\right)\\ \; & =\hat{e}\left(g^{z_{uid}},g^{\lambda_{i}}\right)\hat{e}\left(g^{z_{uid}},g^{\alpha_{\rho (i)}t_{i}}\right)\cdot \hat{e}\left(g^{z_{uid}\alpha_{\theta }},g^{-t_{i}}\right)\hat{e}\left(H_{0}{\left(uid\right)}^{\beta_{\theta }}H_{1}{\left(att_{i}\right)}^{t},g^{-t_{i}}\right)\cdot \\ \; & \;\hat{e}\left(H_{0}\left(uid\right),g^{\beta_{\rho (i)}t_{i}}\right)\hat{e}\left(H_{0}\left(uid\right),g^{w_{i}}\right)\cdot \hat{e}\left(g^{t},H_{1}{\left(\delta \left(i\right)\right)}^{t_{i}}\right)\\ \; & =\hat{e}{\left(g,g\right)}^{z_{uid}\lambda_{i}}\hat{e}{\left(g,g\right)}^{z_{uid}\alpha_{\rho (i)}t_{i}}\cdot \hat{e}{\left(g,g\right)}^{-z_{uid}\alpha_{\theta }t_{i}}\hat{e}\left(H_{0}{\left(uid\right)}^{\beta_{\theta }},g^{-t_{i}}\right)\hat{e}\left(H_{1}{\left(att_{i}\right)}^{t},g^{-t_{i}}\right)\cdot \\ \; & \;\hat{e}\left(H_{0}\left(uid\right),g^{\beta_{\rho (i)}t_{i}}\right)\hat{e}{\left(H_{0}\left(uid\right),g\right)}^{w_{i}}\cdot \hat{e}\left(H_{1}{\left(\delta \left(i\right)\right)}^{t_{i}},g^{t}\right)\\ \; & =\hat{e}{\left(g,g\right)}^{z_{uid}\lambda_{i}}\hat{e}{\left(H_{0}\left(uid\right),g\right)}^{w_{i}}\cdot \left(\hat{e}{\left(g,g\right)}^{z_{uid}\alpha_{\rho (i)}t_{i}}\cdot \hat{e}{\left(g,g\right)}^{-z_{uid}\alpha_{\rho (i)}t_{i}}\right)\cdot \\ \; & \;\left(\hat{e}{\left(H_{0}\left(uid\right),g\right)}^{-t_{i}\beta_{\rho (i)}}\cdot \hat{e}\left(H_{0}\left(uid\right),g^{\beta_{\rho (i)}t_{i}}\right)\right)\left(\hat{e}{\left(H_{1}\left(\delta \left(i\right)\right),g\right)}^{-t_{i}t}\cdot \hat{e}\left(H_{1}{\left(\delta \left(i\right)\right)}^{t_{i}t},g\right)\right)\\ \; & =\hat{e}{\left(g,g\right)}^{z_{uid}\lambda_{i}}\hat{e}{\left(H_{0}\left(uid\right),g\right)}^{w_{i}}. \end{array}$$

Then, Equation (16) is calculated as

(22) $$\begin{array}{cc} tct & =\prod_{i\in I}{\left(tct_{i}\right)}^{c_{i}}\\ \; & =\prod_{i\in I}{\left(\hat{e}{\left(g,g\right)}^{z_{uid}\lambda_{i}}\hat{e}{\left(H_{0}\left(uid\right),g\right)}^{w_{i}}\right)}^{c_{i}}\\ \; & =\prod_{i\in I}\left(\hat{e}{\left(g,g\right)}^{z_{uid}\lambda_{i}c_{i}}\cdot \hat{e}{\left(H_{0}\left(uid\right),g\right)}^{w_{i}c_{i}}\right)\\ \; & =\hat{e}{\left(g,g\right)}^{z_{uid}s}. \end{array}$$

Finally, the calculation process of Equation (18) is $\frac{C_{0}}{tct^{\frac{1}{SK_{uid}}}}=\frac{F\cdot \hat{e}{\left(g,g\right)}^{s}}{{\hat{e}{\left(g,g\right)}^{z_{uid}s}}^{\frac{1}{z_{uid}}}}=F$.

### 6.2. Static Security

**Theorem 1.** 
*In the event that the q-DPBDHE2 problem is difficult, an adversary without probabilistic polynomial-time (PPT) can defeat the BM-ABSE scheme by statically corrupting a collection of attribute authorities.*

**Lemma 1.** 
*Let A be the access matrix constructed using LSSS. The rows of the matrix controlled by the corrupt attribute authorities are denoted as C⊆[l]. The corrupted rows can span a subspace of dimension c. According to the secret shares divided by A, they are the same as those divided by $A^{\prime }$, where $A_{x,j}^{\prime }=0,\forall \left(x,j\right)\in C\times \left[n-c\right]$. This lemma enables the adversary to isolate the matrix rows related to the corrupted attribute authorities. $A=\left[\begin{array}{cccc} A_{1,1} & A_{1,2} & \cdots & A_{1,n}\\ A_{2,1} & A_{2,2} & \cdots & A_{2,n}\\ A_{3,1} & A_{3,2} & \cdots & A_{3,n}\\ \vdots & \vdots & \ddots & \vdots \\ A_{\ell ,1} & A_{\ell ,2} & \cdots & A_{\ell ,n} \end{array}\right]⇝A^{\prime }=\left[\begin{array}{c} \begin{array}{cccccc} 0 & \cdots & 0 & A_{1,n-c+1}^{\prime } & \cdots & A_{1,n}^{\prime }\\ A_{2,1}^{\prime } & \cdots & A_{2,n-c}^{\prime } & A_{2,n-c+1}^{\prime } & \cdots & A_{2,n}^{\prime }\\ 0 & \cdots & 0 & A_{3,n-c+1}^{\prime } & \cdots & A_{3,n}^{\prime }\\ \vdots & \ddots & \vdots & \vdots & \ddots & \vdots \\ A_{\ell ,1}^{\prime } & \cdots & A_{\ell ,n-c}^{\prime } & A_{\ell ,n-c+1}^{\prime } & \cdots & A_{\ell ,n}^{\prime } \end{array} \end{array}\right]$*

**Proof.**  Suppose there exists an adversary A that can break the BM-ABSE scheme with advantage ε in the static security model. The *q*-DPBDHE2 problem can be resolved by constructing a simulator B that is based on A.

1. *Init*: B generates public parameters PP using the (D,T) of *q*-DPBDHE2 problem challenger and sends them to A.
2. *Query–Attacker*: A specifies a collection of compromised attribute authorities denoted as $C_{AAs}$, uncorrupted attribute authorities denoted as $N_{AAs}$, already queried attribute sets $Q_{a}={\left\{uid_{i},Suid_{i}\right\}}_{i=1}^{m}$, two plaintexts of the same length $m_{0},m_{1}\in G_{T}$, and the access policy $\left(A^{*},\delta^{*}\right)$ to be challenged. $H_{0}$ and $H_{1}$ are represented as random oracles, and A specifies the identity set $Q_{H_{0}}$ for querying $H_{0}$ and the attribute set $Q_{H_{1}}$ for querying $H_{1}$.
3. *Query–Public Key of Attribute Authorities*: B converts A to $A^{\prime }$, where $n^{\prime }=n-c$, and responds to A’s query for the public key of the attribute authorities as follows:
  - If the queried attribute authority is not in the access policy $\left(A^{*},\delta^{*}\right)$, that is, aid∉ρ[ℓ], then B sends $MPK_{aid}=\left\{g^{\alpha_{aid}},g^{\beta_{aid}}\right\}$ to A, where $\alpha_{aid},\beta_{aid}\in Z_{p}^{*}$.
  - If the queried attribute authority is in the access policy $\left(A^{*},\delta^{*}\right)$ and its managed matrix rows are denoted as I={i∈[ℓ]}, then B randomly selects $\alpha_{aid}^{\prime },\beta_{aid}^{\prime }\in Z_{p}^{*}$ and sets
    (23) $$\left\{\begin{array}{c} a_{aid}=\alpha_{aid}^{\prime }+\sum_{i\in I}b_{i}a^{q+1}A_{i,1}^{\prime },\\ b_{aid}=\beta_{aid}^{\prime }+\sum_{i\in I}\sum_{j=2}^{n^{\prime }}b_{i}a^{q+2-j}A_{i,j}^{\prime }. \end{array}\right.$$
  Finally, B sends $MPK_{aid}=\left\{g^{\alpha_{aid}},g^{\beta_{aid}}\right\}$ to A.
4. *Query–$H_{0}\left(Q_{H_{0}}\right)$*: Depending on the different conditions satisfied by the queried identity identifier and attribute set, B responds to A’s query in the following situations:
  - If $uid_{i}\notin Q_{a}$ and $uid_{i}\in Q_{H_{0}}$, B responds to A’s query with a random element from the group G.
  - If $uid_{i}\notin Q_{a}$ and $S_{uid_{i}}\notin \left(A^{*},\delta^{*}\right)$, B randomly selects ${\tilde{h}}_{i}\in Z_{p}^{*}$ and sets the following:
    (24) $$H_{0}\left(uid_{i}\right)=g^{{\tilde{h}}_{i}}g^{\sum_{k=2}^{n^{\prime }}a^{k-1}}.$$
  - If $uid_{i}\notin Q_{a}$ and $S_{uid_{i}}\in \left(A^{*},\delta^{*}\right)$, there exists a set of matrix rows $I^{\prime }\subseteq \left[\ell \right]$ such that $\delta \left(i^{\prime }\right)\in S_{uid_{i}}$, and this set of matrix rows can be seen as managed by the corrupted attribute authorities. There exists a vector $d_{i}\in Z_{p}^{1\times n}$ with the first element being 0 satisfying $A_{x}^{\prime }\cdot d_{i}=0$. In addition, this vector is orthogonal to the corrupted matrix rows. B chooses a random element ${\tilde{h}}_{i}\in Z_{p}^{*}$ and sets the following:
    (25) $$H_{0}\left(uid_{i}\right)=g^{{\tilde{h}}_{i}}g^{\sum_{k=2}^{n^{\prime }}\left(a^{k-1}\cdot d_{i,k}\right)}.$$
  Finally, B sends $H_{0}\left(uid_{i}\right)$ to A.
5. *Query–$H_{1}\left(Q_{H_{1}}\right)$*: Let aid be an attribute authority managing an attribute in $Q_{H_{1}}$. B responds to A’s query based on the following:
  - If aid∉ρ[ℓ] or $aid\in C_{AAs}$, B responds to A’s query with a random element from the group G.
  - If aid∈ρ[ℓ], then the matrix rows controlled by this attribute authority are denoted as $I^{\prime \prime }=\left\{i|\rho \left(i\right)=aid\right\}\setminus \left\{i|\delta \left(i\right)=att_{i}\right\}\subseteq \left[\ell \right]$. B randomly selects ${\tilde{h}}_{i}\in Z_{p}^{*}$ and sets the following:
    (26) $$H_{1}\left(att_{i}\right)=g^{{\tilde{h}}_{att_{i}}}g^{\sum_{i\in I^{\prime \prime }}\sum_{j\in [n^{\prime }]}\left(b_{i}a^{q+1-j}\cdot A_{i,j}^{\prime }\right)}.$$
  Finally, B sends $H_{1}\left(att_{i}\right)$ to A.
6. *Query–Attribute Decryption Key*: A submits an attribute set $\left(uid_{i},S_{uid_{i}}\right)$ to B. For an attribute $att_{i}\in S_{uid_{i}}$, B responds to A’s query according to the following situations:
  (a) If the attribute authority of attribute $att_{i}$ is not in the access policy $\left(A^{*},\delta^{*}\right)$, B randomly selects $\alpha_{aid},\beta_{aid},t\in Z_{p}^{*}$ and outputs the following:
    (27) $$\left\{\begin{array}{c} K_{uid_{i},att_{i}}=g^{z_{uid_{i}}\alpha_{aid}}H_{0}{\left(uid_{i}\right)}^{\beta_{aid}}H_{1}{\left(att_{i}\right)}^{t},\\ L_{uid_{i},att_{i}}=g^{t}. \end{array}\right.$$
  (b) If the attribute authority of attribute $att_{i}$ is in the access policy $\left(A^{*},\delta^{*}\right)$ but $S_{uid_{i}}\notin \left(A^{*},\delta^{*}\right)$, the matrix rows controlled by this attribute authority are denoted as $I^{\prime }=\left\{i|\rho \left(i\right)=aid\right\}\subseteq \left[\ell \right]$. B sets $t=-\sum_{k\in [n^{\prime }]}a^{k}$ and computes the following:
    (28) $$\left\{\begin{array}{c} K_{uid_{i},att_{i}}=g^{uid_{i}}H_{0}{\left(uid_{i}\right)}^{\sum_{i\in I^{\prime }}\sum_{j=2}^{n^{\prime }}b_{i}a^{q+2-j}A_{i,j}^{\prime }}g^{\alpha_{aid}^{\prime }}\cdot \\ \;\;\;\;\;H_{1}{\left(att_{i}\right)}^{t}g^{\sum_{i\in I^{\prime }}b_{i}a^{q+1}A_{i,1}^{\prime }}H_{0}{\left(uid_{i}\right)}^{\beta_{aid}^{\prime }},\\ L_{uid_{i},att_{i}}=g^{-\sum_{k\in [n^{\prime }]}a^{k}}. \end{array}\right.$$
  (c) If the attribute authority of attribute $att_{i}$ is in the access policy $\left(A^{*},\delta^{*}\right)$ and $S_{uid_{i}}\in \left(A^{*},\delta^{*}\right)$, B sets $t=-\sum_{k\in [n^{\prime }]}a^{k}d_{i,k}$ and calculates the following:
    (29) $$\left\{\begin{array}{c} K_{uid_{i},att_{i}}=g^{uid_{i}}H_{0}{\left(uid_{i}\right)}^{\sum_{i\in I^{\prime }}\sum_{j=2}^{n^{\prime }}b_{i}a^{q+2-j}A_{i,j}^{\prime }}g^{\alpha_{aid}^{\prime }}\cdot \\ \;\;\;\;\;H_{1}{\left(att_{i}\right)}^{t}g^{\sum_{i\in I^{\prime }}b_{i}a^{q+1}A_{i,1}^{\prime }}H_{0}{\left(uid_{i}\right)}^{\beta_{aid}^{\prime }},\\ L_{uid_{i},att_{i}}=g^{-\sum_{k\in [n^{\prime }]}a^{k}d_{i,k}}. \end{array}\right.$$
7. *Query–Ciphertext*: B calculates $C_{0}=m_{b}\cdot T,b\in \left\{0,1\right\}$; then, it constructs two vectors $v=\left(sa^{q+1},0,\ldots ,0\right),\vec{w}=\left(0,sa^{q},\ldots ,sa^{q-n^{\prime }+2},0,\ldots ,0\right)$ and computes the ciphertext components based on the following situations:
  (a) For the matrix rows $i^{*}$ controlled by the corrupted attribute authority in the access structure $\left(A^{*},\delta^{*}\right)$, there exists $\lambda_{i^{*}}=0$, $w_{i^{*}}=0$. B computes the corresponding ciphertext components:
    (30) $$\left\{\begin{array}{c} C_{1,i^{*}}=g^{\alpha_{\rho (i^{*})}t_{i^{*}}},\\ C_{2,i^{*}}=g^{\beta_{\rho (i^{*})}t_{i^{*}}},\\ C_{3,i^{*}}=g^{-t_{i^{*}}},\\ C_{4,i^{*}}=H_{1}{\left(\delta \left(i^{*}\right)\right)}^{t_{i^{*}}}. \end{array}\right.$$
  (b) For the normal matrix rows $i^{*}$ in the access structure $\left(A^{*},\delta^{*}\right)$, there exists $\lambda_{i^{*}}=sa^{q+1}\cdot A_{i^{*},1}^{\prime },w_{i^{*}}=\sum_{j=2}^{n^{\prime }}sa^{q+2-j}A_{i^{*},j}^{\prime }$. B computes the corresponding ciphertext components:
    (31) $$\left\{\begin{array}{c} C_{1,i^{*}}={\left(g^{\sum_{i\in I\setminus \{i^{*}\}}sb_{i}a^{q+1}/b_{i^{*}}}\right)}^{-A_{i^{*},1}^{\prime }},\\ C_{2,i^{*}}={\left(g^{\sum_{i\in I\setminus \{i^{*}\}}\sum_{j=2}^{n}sb_{i}a^{q+2-j}/b_{i^{*}}}\right)}^{-A_{i^{*},j}^{\prime }},\\ C_{3,i^{*}}=g^{-t_{i^{*}}}=g^{s/b_{i^{*}}},\\ C_{4,i^{*}}={\left(g^{\sum_{i\in I^{\prime \prime }}\sum_{j\in [n^{\prime }]}sb_{i}a^{q+1-j}/b_{i^{*}}}\right)}^{-A_{i^{*},j}^{\prime }}. \end{array}\right.$$
8. *Guess*: A outputs its guess $b^{\prime }$. If $b^{\prime }=b$, then B returns $T=\hat{e}{\left(g,g\right)}^{sa^{q+1}}$; otherwise, T=R. According to the security game rules mentioned above, A cannot easily compute $\hat{e}{\left(g,g\right)}^{sa^{q+1}}$ based on the known information. Although $g^{sa^{q+1}b_{j}/b_{j^{\prime }}}$ is known, the *q*-DPBDHE2 problem remains difficult since $j\neq j^{\prime }$.

□

### 6.3. IND-CKA Security

**Theorem 2.** 
*Adversaries who lack probabilistic polynomial-time (PPT) are capable of defeating the BM-ABSE scheme through a chosen-keyword attack if the DBDH problem is difficult.*

**Proof.**  Suppose that the BM-ABSE scheme proposed in the IND-CKA security model can be breached by an adversary A with an advantage of ε. The DBDH problem can be resolved by constructing a simulator B that is based on A.

1. *Init*: The simulator B generates public parameters and transmits them to the attacker A.
2. *Phase 1*: The attacker A performs a trapdoor search query by submitting a collection of keywords $KW^{\prime }=\left\{kw_{1}^{\prime },\ldots ,kw_{d}^{\prime }\right\}$ to the simulator B. The simulator B selects a random element $\eta \in Z_{p}^{*}$ and runs the TrapGen to create a search trapdoor that is linked to $KW^{\prime }$:
  (32) $$Td=(T_{1}=g^{\eta z_{uid}},T_{2}=g^{\gamma \eta z_{uid}},T_{3}=\prod_{i=1}^{d^{\prime }}g^{\gamma H(kw_{i}^{\prime })}).$$
3. *Challenge*: The simulator B is sent two keyword sets $KW_{0}^{\prime },KW_{1}^{\prime }$ by the attacker A that are not interrogated in *Phase 1*. The simulator B then runs the KeywordEncrypt to arbitrarily encrypt a keyword set in $KW_{b}^{\prime },b\in \left\{0,1\right\}$.
4. *Phase 2*: The attacker A can continue the query in *Phase 1*, but it is prohibited from querying the search trapdoor of $KW_{0}^{\prime }$ or $KW_{1}^{\prime }$.
5. *Guess*: The attacker A outputs its conjecture $b^{\prime }$. If $b^{\prime }=b$, the adversary A prevails in the aforementioned security game. The adversary A has an advantage of ε/2 in differentiating between $g^{\varphi }$ and $g^{\gamma H(kw_{0})\tau }$ if it can succeed in the IND-CKA security game with an advantage of ε, where $\varphi \in Z_{p}^{*}$. The advantage of the attacker A in differentiating between $g^{\gamma H(kw_{0})\tau }$ and $g^{\gamma H(kw_{1})\tau }$ is tantamount to the benefit of differentiating between $g^{\varphi }$ and $g^{\gamma \tau }$. Due to the difficulty of the DBDH problem, the advantage ε can be neglected.

□

## 7. Performance and Evaluation

### 7.1. Functionality Comparison

Table 2 presents a functional comparison between the proposed BM-ABSE scheme and existing similar schemes. Similar to references [22,23], our scheme has multi-authority to decentralize management responsibilities and avoid single points of failure. All of these schemes are based on cloud storage, making them suitable for large-scale data scenarios in IoT. In addition, like only a few schemes, we support multi-keyword search to improve query efficiency. By integrating blockchain technology and smart contracts, our scheme ensures the integrity and verifiability of data as well as the credibility of search results. Finally, whereas most schemes guarantee IND-CKA security, we are the only one that formally proves static security.

### 7.2. Storage Overhead

Table 3 illustrates the storage cost analysis of the our scheme. In the BM-ABSE scheme, the attribute decryption key size is $2N_{u}\left|G\right|$, aligning with schemes that feature a single attribute authority such as [24,27]. Except for scheme [26], the storage overhead of the data index in the other approaches is linearly related to the number of keywords defined by the data owner. Meanwhile, the data index storage overhead of scheme [27] is the same as our scheme and exceeds the other schemes to achieve the optimum. Scheme [26] integrates access control into the search algorithm, which leads to an increase in its data index storage overhead with the quantity of attributes, and each search procedure is capable of matching only one keyword at a time. Moreover, only our scheme and scheme [27] have a constant storage overhead for search trapdoors. In schemes [24,26], the size of the search trapdoor exhibits a linear relationship with the quantity of keywords or attributes, leading to a substantial increase in storage burden as the number of keywords increases. Every comparison approach has a constant storage overhead for the partially decrypted ciphertext. Specifically, the partially decrypted ciphertext consists of two ciphertext components within the group $G_{T}$: one computed by the cloud platform and the other derived from the original ciphertext.

### 7.3. Computational Overhead

This section provides a theoretical analysis of the computational overhead of the BM-ABSE scheme based on Table 4. The scheme [22] has the same exponential computational overhead as our scheme in the ADKGen phase. This is attributed to the fact that both schemes employ multiple attribute authorities to oversee the attributes in the system; compared with the single attribute authority scheme, there is a slightly higher computational overhead. However, the setting of multiple attribute authorities is more aligned with practical requirements and avoids performance bottlenecks caused by single points of failure. Scheme [27] has the same exponential computational overhead as the proposed scheme during KeywordEncrypt. In particular, scheme [27] requires more hash computations in the stage of TrapGen. Since hash operations are less computationally expensive than exponential operations and bilinear pairings, they are not included in the table. The BM-ABSE scheme requires only (4k+1)E exponential operations in the DataEncrypt phase. All comparison schemes delegate the intricate decryption computations to the cloud. The proposed scheme reduces the computational load on the user end to just one exponential operation by outsourcing (4k)P bilinear pairing computation to the cloud.

### 7.4. Experimental Analysis

The development and operational stack comprises Go 1.21.6, Python 3.11.5, JDK 17.0.12, MySQL 5.7.26, Solidity 0.8.24, and Docker 27.5.1. To evaluate the performance of the BM-ABSE scheme, this section assesses the efficiency of the main algorithms under three security levels: 70-bit security, 80-bit security, and 90-bit security. The Charm framework utilizes three elliptic curves (MNT159, SS512, and MNT201) to achieve different security levels. Among them, SS512 is used for symmetric bilinear pairing, while MNT159 and MNT201 are used for asymmetric bilinear pairing. The simulation environment is configured as follows:

- OS: Ubuntu 22.04 64-bit (6.8.0-60-generic) (Canonical Ltd., London, UK);
- CPU: Intel(R) Core(TM) i5-12500H @ 2.50 GHz (Intel Corporation, Santa Clara, CA, USA);
- RAM: Samsung DDR4-3200 16 GB (Samsung Electronics Co., Ltd., Suwon, Republic of Korea);
- MB: HONOR GLO-FX6-PCB (M1020)/BIOS 1.13 (Honor Device Co., Ltd., Shenzhen, China);
- HDD: Samsung SSD 980 PRO 1 TB (Samsung Electronics Co., Ltd., Suwon, Republic of Korea).

**Figure 2:**
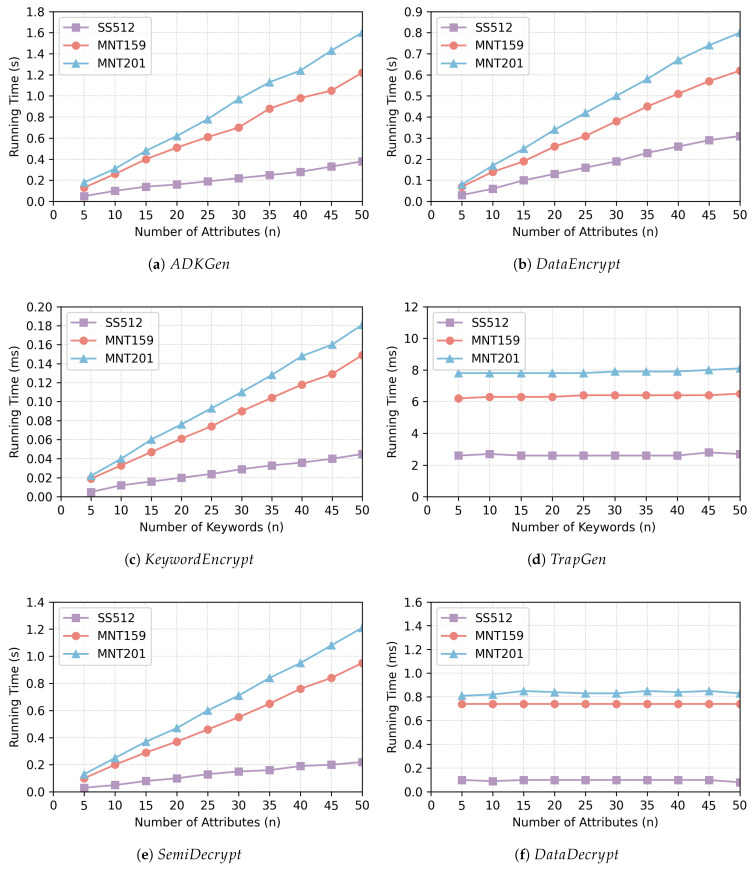
The running time of the algorithm at each stage under different security levels.

Table 5 summarizes the time cost of some basic cryptographic operations in this configuration. In addition, the execution time of the algorithm at each stage under different security levels is shown in Figure 2. As illustrated in Figure 2a, the computational overhead for generating the attribute decryption key increases linearly with the number of user attributes, which corroborates the theoretical analysis above. The time-consuming computational operations in the ADKGen algorithm are mainly exponential operations. Under the SS512 setting, ADKGen exhibits the fastest computation. Although the execution time is slightly higher than that of SS512 in asymmetric bilinear pairing settings (MNT159 or MNT201), it remains practical for real-world IoT scenarios. The execution duration of the KeywordEncrypt process is illustrated in Figure 2c, and the time overhead is approximately linear with the quantity of keywords collected by the data owner. As the number of keywords increases, the running time increases accordingly. Under the three security levels, the index construction time for 50 keywords remains within 0.2 ms, which indicates that the algorithm is still efficient even in resource-constrained application scenarios.

As observed in Figure 2d,f, the execution time of the TrapGen algorithm and the DataDecrypt algorithm remains almost constant with the increasing quantity of keywords or attributes at different security levels. However, it should be noted that the performance of both algorithms remains influenced by the quantity of keywords or attributes. TrapGen and DataDecrypt encompass multiplication and hash operations, but these contribute negligibly to the overall running time. For different security levels, the average execution time of the DataDecrypt algorithm is less than 1 ms. According to Figure 2b,e, the execution times of the DataEncrypt algorithm and the SemiDecrypt algorithm both increase gradually as the quantity of attributes grows. For DataEncrypt, the encryption time for the plaintext is 0.31 s with SS512, 0.62 s with MNT159, and 0.80 s with MNT201 when the quantity of attributes is 50. For each of the three security levels, the time stays below 1 s. For SemiDecrypt, the maximum execution time for 50 attributes is 1.2 s, which does not present a computational burden for a powerful cloud server.

**Figure 3:**
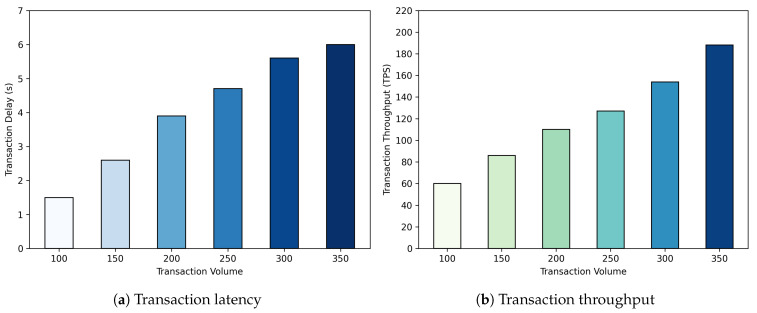
Simulation result.

The BM-ABSE scheme implements the SearchMatch algorithm using the Solidity smart contract language and deploys it on the blockchain. To further evaluate the efficiency of the chain code, we set up a blockchain network, configured with four peer nodes and two sorting nodes, using Hyperledger Fabricand. In addition, we measure transaction latency and throughput using Hyperledger Caliper, and the results are shown in Figure 3. As shown in Figure 3a, transaction latency increases almost linearly with rising transaction volume. This is a typical phenomenon of distributed consensus systems, as transactions must queue at nodes to await processing and consensus. Figure 3b shows that the actual network throughput increases linearly with the transmission rate and reaches a plateau at nearly 200 TPS, indicating that the network resources are fully utilized. Under the current test of a four-node network configuration, this is the maximum performance limit that our system can handle. These results confirm that the logic of the SearchMatch algorithm is efficient. The dominant performance bottleneck stems from the processing capacity of the underlying Hyperledger Fabric platform under specific configurations. The experiment provides a benchmark for the performance of the system at a defined scale, demonstrating predictable scalability as load increases.

In an extreme massive IoT scenario, tens of thousands of terminals need to be aggregated. If each endpoint triggers one search transaction every five minutes, the transaction throughput could reach thousands of TPS. However, not all IoT terminals require frequent data search operations in practice. Monitoring devices primarily read data, and search requests are usually initiated by upper-layer applications at a frequency far lower than that of data reporting. Consequently, the average load remains at the hundreds-of-TPS level. Moreover, the proposed scheme distributes the most time-consuming search matching computation and result verification to multiple blockchain nodes for parallel execution through smart contracts, eliminating the single-point performance bottleneck of centralized servers, making it fundamentally more scalable. In summary, although the absolute throughput measured in this experiment on a single test network is limited, the architecture and methodology proposed in this paper are applicable to intelligent systems.

## 8. Conclusions

With the exponential growth of IoT data and the increasing demand for cross-domain collaboration, how to achieve reliable data sharing between resource-constrained terminals and untrusted clouds has become a core challenge in the deeper development of intelligent systems. In this paper, we propose a blockchain-based multi-authority IoT data-sharing scheme with attribute-based searchable encryption for intelligent system (BM-ABSE), aiming to address the issues of security, efficiency, and verifiability of data sharing in the IoT environment. This scheme adopts attribute-based searchable encryption, multi-authority mechanism, and blockchain technology to protect the confidentiality of data, achieve fine-grained access control, and guarantee the efficiency of the search and credibility of the search results. At the same time, by matching search trapdoors and data indexes on the blockchain through smart contracts, it can effectively prevent unauthorized or malicious third parties from returning biased or commercial search results. Given the resource constraints of IoT devices, partially decryption calculations can be directly completed in the cloud without the need for additional computational outsourced keys. Theoretical analysis indicates that BM-ABSE satisfies both static security and IND-CKA security under the standard model and can effectively resist the stealing or tampering of ciphertexts and keywords by attackers. Even if attackers statically corrupt any number of attribute authorities after obtaining public parameters, they still cannot obtain any additional information about plaintexts or keywords from the ciphertexts or indexes. Experimental simulation shows that our scheme has good computational efficiency on IoT devices with limited resources. The running time of the core algorithms maintains in milliseconds, and it has linearly controllable overhead as the number of attributes increases. Under the three security levels, the terminal only needs one exponential operation to complete the final decryption, which significantly alleviates the computation and energy consumption bottlenecks of IoT devices. The overall running time of the core algorithms remains in milliseconds. Among them, the time consumption of ADKGen, DataEncrypt, and SemiDecrypt algorithms increases linearly with the number of attributes, but it still less than 1 s under the condition of 50 attributes, which makes it scalable for large-scale IoT deployment. In addition, when the transaction volume increases to 350, the throughput of smart contracts on the blockchain increases to nearly 200 TPS, indicating that the system can still maintain a certain degree of scalability when handling a large number of transactions. Finally, introducing revocable and dynamic attribute update mechanisms to support key rotation and permission recovery throughout the entire lifecycle of IoT devices is a future research direction.

## Figures and Tables

**Table 1:** The symbols employed throughout this paper and their corresponding definitions.

Notation	Definition
PP	Public parameters
GID	Global identifiers
AID	Identifiers of attribute authorities
UID	Identifiers of data user and data owner
*U*	Attribute universe
*S*	Attribute set
$S_{uid,aid}$	Attribute set managed by the attribute authority aid belongs to the data user uid
$MPK_{\theta },MSK_{\theta }$	Master secret key pair of authority θ
$ADK_{uid,aid}$	Attribute decryption key for user uid from authority aid
CT	Ciphertext of the IoT data
Addr	Storage address
$I_{dx}$	Data index
Td	Search trapdoor generated by data user
SCT	Semi-decrypted ciphertext generated by cloud
(A,δ)	The data access policy
δ(x)	A function that maps a row of matrix A to an attribute
ρ(x)	A function that maps a row of matrix A to an attribute authority
$N_{u}$	The quantity of user attributes
*k*	The quantity of attributes in the access policy
$d,d^{\prime }$	The quantities of keywords derived from the plaintext and contained in search trapdoor
E,P	The exponential operations on groups and bilinear pairing operations
$\left|G|,\right|G_{T}\left|,\right|Z_{p}|$	The lengths of elements in group G, $G_{T}$ and $Z_{p}$

**Table 2:** Functionality comparison.

	[22]	[24]	[25]	[26]	[27]	[23]	[28]	Ours
Attribute authority	multiple	single	single	single	single	multiple	single	multiple
Access structure	LSSS	Tree	AND	LSSS	LSSS	LSSS	LSSS	LSSS
Multiple keywords	✓	✓	×	×	✓	×	×	✓
Results verification	×	×	×	×	✓	×	✓	✓
Blockchain	×	×	×	×	✓	✓	✓	✓
Data storage	cloud	cloud	cloud	cloud	cloud	cloud	cloud	cloud
Security model	IND-CPA, IND-CKA	IND-CPA, IND-CKA	IND-CP-CKA	ACKSA	IND-CKA	CKA	CKA	static, IND-CKA

**Table 3:** Storage overhead comparison.

	[24]	[26]	[27]	Ours
Attribute decryption key	$\left(2N_{u}+4)|G\right|$	$\left(N_{u}+3)|G\right|$	$\left(2N_{u}+1)|G\right|$	$2N_{u}\left|G\right|$
Data index	$\left(2d\right)|G|+|G_{T}|$	$\left(2N_{u}+2)|G\right|$	(d+3)|G|	(d+3)|G|
Search trapdoor	$\left(2d^{\prime }+1)|G\right|$	$\left(N_{u}+2)|G\right|$	3|G|	3|G|
Partially decrypted ciphertext	$2|G_{T}|$	-	$2|G_{T}|$	$2|G_{T}|$
Ciphertext	$\left(2k+2d+2\right)|G|+2|G_{T}|$	$\left(4k+2\right)|G|+2|G_{T}|$	$\left(3k+2\right)|G|+|G_{T}|$	$4k|G|+|G_{T}|$

**Table 4:** Computational overhead comparison.

	[22]	[24]	[26]	[27]	Ours
ADKGen	$(4N_{u})E$	$(2N_{u}+6)E$	$(N_{u}+3)E$	$(2N_{u}+4)E$	$(4N_{u})E$
KeywordEncrypt	$\left(2N_{u}+7\right)E+\left(2N_{u}+1\right)P$	(2d)E	$(3N_{u}+2)E$	(d+3)E	(d+3)E
TrapGen	$(N_{u}+1)E+P$	$(2N_{u}+d^{\prime })E$	$(N_{u}+2)E$	3E	3E
SearchMatch	$(2N_{u}+4)E+3P$	$2E+(N_{u}\cdot d^{\prime }+1)P$	(2k+1)P	$(d^{\prime }+2)P$	4P
DataEncrypt	-	(2k+4)E+2P	(6k+4)E	(4k+3)E+(k)P	(4k+1)E
SemiDecrypt	(4k)P	(k+2)P	-	(3k)E	(4k)P
DataDecrypt	2E	*P*	(2k+1)P	E+P	*E*

**Table 5:** The overhead of basic calculations.

Operation	SS512 (ms)	MNT159 (ms)	MNT201 (ms)
$E(G_{1})$	0.85806	0.31256	0.43513
$E(G_{2})$	0.86236	2.92539	3.67335
$E(G_{T})$	0.08363	0.70905	0.82113
*P*	0.63634	2.25567	2.70392

## Data Availability

The original contributions presented in the study are included in the article, and further inquiries can be directed to the corresponding author.
